# Supplementary figures and images for: Aquaporin-3 regulates endosome-to-cytosol transfer via lipid peroxidation for cross presentation
Source: PLoS One. 2020 Nov 24;15(11):e0238484. doi: 10.1371/journal.pone.0238484 (PMC7685505; doi:10.1371/journal.pone.0238484)

# Supplementary Figure 1

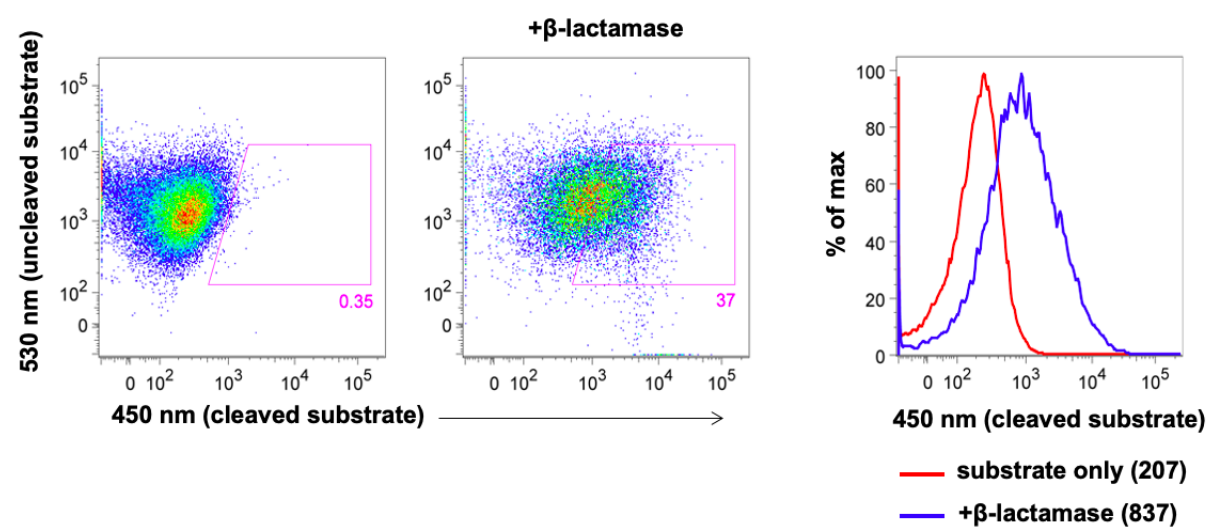

Supplement: S1 Fig — Cleaved substrate is the median fluorescence intensity of the 450 nM channel. (PDF) [file pone.0238484.s001.pdf]

# Supplementary Figure 2

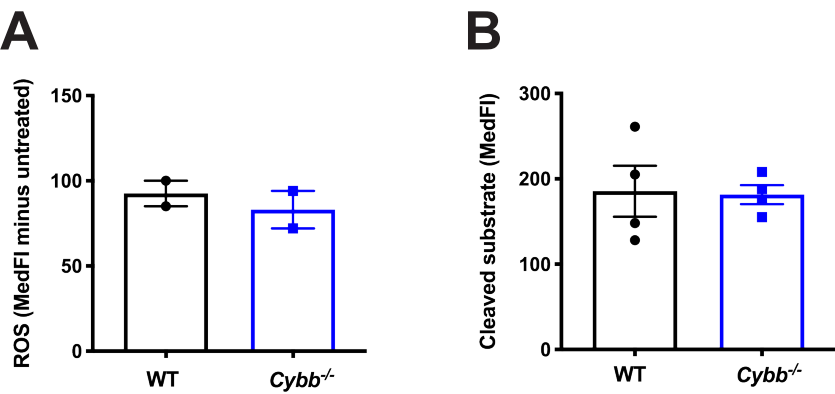

Supplement: S2 Fig — (A) Cybb-/- or WT BMDCs were loaded with the ROS sensor CM-H2DCFDA followed by incubation with 100 μg/ml β-lactamase. (B) ECT was evaluated with 100 μg/ml β-lactamase. (PDF) [file pone.0238484.s002.pdf]

# Supplementary Figure 3

**A**

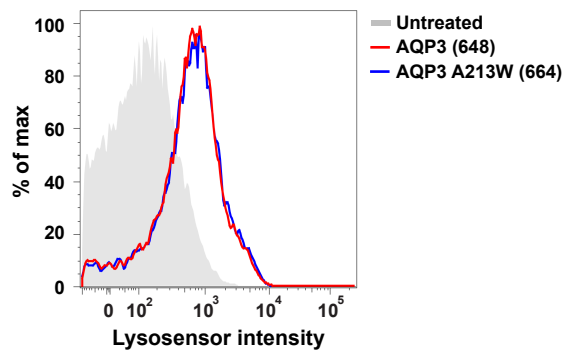

**B**

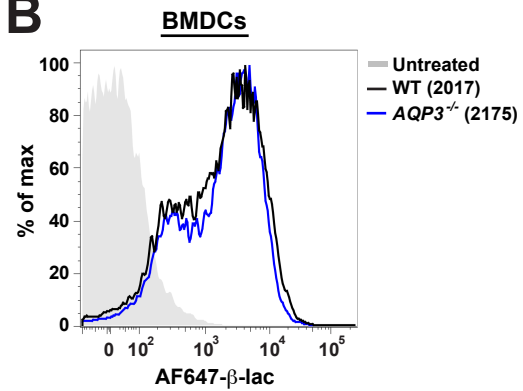

**C**

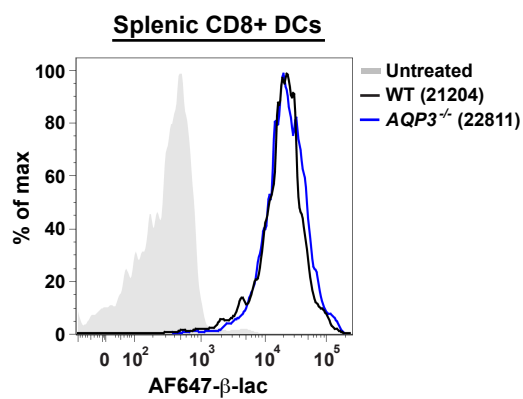

**D**

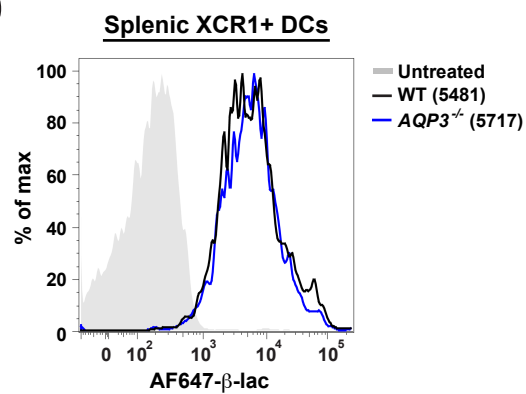

Supplement: S3 Fig — (A) Transfected and sorted HEK293s were incubated with LysoSensor Green DND-189 for 30 minutes followed by analysis by flow cytometry. The greater the LysoSensor fluorescence intensity, the lower the pH. MedFI is displayed. (B) Phagocytosis of 50 μg/ml fluorescently-labeled β-lactamase in BMDCs from AQP3-/- or WT control mice. (C) Phagocytosis of 0.5 mg/ml fluorescently-labeled β-lactamase in splenic CD11c+CD8+ DCs isolated from AQP3-/- or WT control mice. (D) Phagocytosis of 0.5 mg/ml fluorescently-labeled β-lactamase in splenic CD11c+XCR1+ DCs isolated from AQP3-/- or WT control mice. MedFI is displayed. (PDF) [file pone.0238484.s003.pdf]

## Supplementary Figure 4

**A**

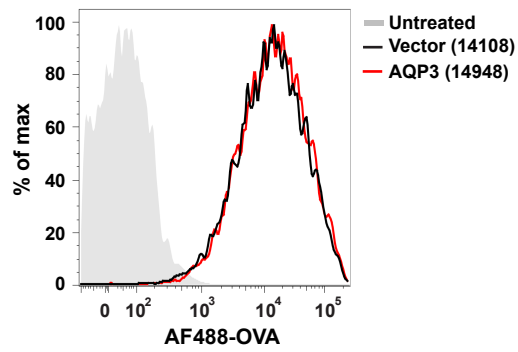

**B**

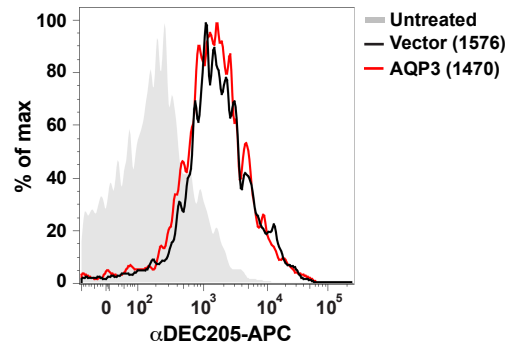

**C**

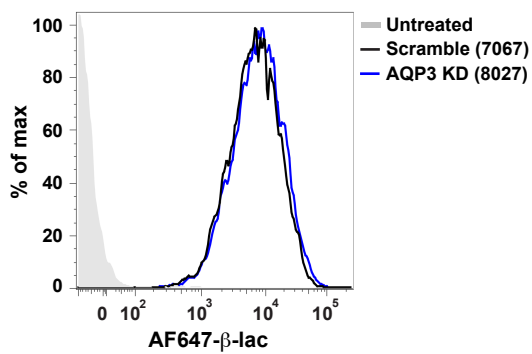

**D**

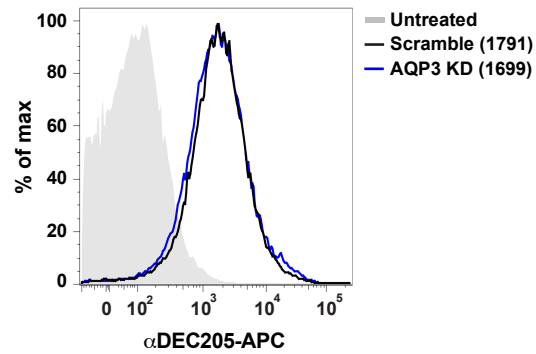

**E**

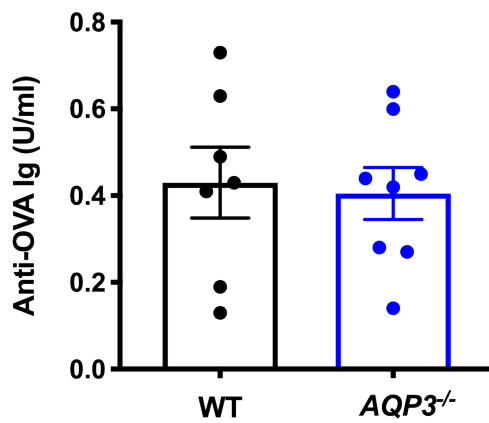

Supplement: S4 Fig — (A) Phagocytosis of 50 μg/ml fluorescently-labeled OVA. (B) Phagocytosis of 1 μg/ml fluorescently-labeled αDEC205 (protocol described in Methods). (C) Phagocytosis of 100 μg/ml fluorescently-labeled β-lactamase. (D) Phagocytosis of 1 μg/ml fluorescently-labeled αDEC205. MedFI is displayed. (E) AQP3-/- or WT control mice were injected i.p. with OVA in CFA. 10 days later, serum was collected and analyzed for anti-OVA Igs. (PDF) [file pone.0238484.s004.pdf]
