# Supplementary material for: Aquaporin-3 regulates endosome-to-cytosol transfer via lipid peroxidation for cross presentation
Source: PLoS One. 2020 Nov 24;15(11):e0238484. doi: 10.1371/journal.pone.0238484 (PMC7685505; doi:10.1371/journal.pone.0238484)
Supplement: Attachment — Submitted filename: Nalle RESPONSE TO REVIEWERS.docx [file pone.0238484.s007.docx]

We thank the reviewers for their positive and constructive comments. Below, please find our point by point responses to the reviewers.

Reviewer #1: In this work, the authors address the mechanisms of endosome-to-cytosol transfer of antigen, and identify Aquaporin-3 as a molecule involved in this process.
While the study is well conducted and the conclusions well supported by the data shown, there are a few points to improve before the manuscript is suitable for publication.

1. The number of independent experiments performed must be indicated in all figures, and the authors need to indicate what is represented (mean ? median ?) and what errors bars are (SEM ? SD ?).

The figure legends have been updated to contain this information.

2. In figure 1C, it would help to add a legend on the volcano plot (what is represented on the left hand side ? right hand side ?).

This figure now displays what is represented on the left- and right-hand sides. The left side is decreased in the ECT+ population, and the right side is increased in the ECT+ population.

3. In figure 1F, it is not clear how ECT is measured. This needs to be better explained in the text.

This experiment is now explained more clearly in the text (page 6, middle paragraph, of the revised manuscript)

4. It is very difficult to understand what is shown in figure 2E. The authors need to better explain this experiment in the text. The legend for the y axis of the plots is also missing.

The text describing this experiment has been updated to contain a more detailed description (page 9, first paragraph). Regarding the figure, the y-axis is now labeled as dsRed.

5. In figure 3A, the authors should show a representative example of FACS data. It would also help understand how the “cleaved substrate” metrics is measured.

Please refer to Response to Reviewers Figure 1 below for a representative example of FACS data. Cleaved substrate is the median fluorescence intensity of the 450 nM channel. We have also now included this figure as a new Supplemental Figure 1 in the revised manuscript.

6. In figure 3F, it is not clear how the authors can conclude on the levels of H2O2 in the phagosomes. Do they assume that the sensor which is added in the medium only reaches the phagosomes of the cells ?

rHyper is a soluble recombinant protein that is added to the media at the start of the experiment, taken up by endocytosis, and its delivery is therefore restricted to phagosomes and other endocytic organelles.

7. In figure 4A, it is difficult to understand what is represented. The authors should show a representative example of FACS data.

In Figure 4, phagosomal lipid peroxidation was calculated by co-incubating bone marrow-derived dendritic cells (BMDCs) with fluorescently-labeled OVA-coated 1 μm Dynabeads and 10 μM C11-bodipy lipid peroxidation indicator. Cells were then homogenized and analyzed by flow cytometry. A representative FACS plot is shown below in Response to Reviewers Figure 2. The phagosomal lipid peroxidation ratio is determined by gating on OVA+ beads (left panel of Figure 2) and then calculating the C11-bodipy ratiometric fluorescent shift indicative of oxidation (represented by an increase in the FITC signal and a decrease in the PE signal).

8. In figure 4D, the authors should indicate in the legend at what time point the cell proliferation was analyzed.

The figure legend now contains the time point at which cell proliferation was analyzed, which is 64 hours after co-culture.

9. In figure 5, it is not clear to me why the authors analyze CD8+ DC and XCR1+ DC separately. These cells both represent cDC1. How did they separate them ? The gating strategy should be shown.

The reviewer is correct that both CD8+ and XCR1+ DCs are commonly classified as cDC1. However, there is data to suggest that the XCR1+ subset may represent those DCs most adept at antigen cross presentation. We therefore hypothesized that CD8+ and XCR1+ splenic dendritic cells (DCs) might display differential sensitivities to genetic inhibition of AQP3. Each subtype was analyzed separately in independent studies, with initial experiments looking at CD8+ DCs and subsequent experiments focused on XCR1+ DCs. The gating strategy is shown below, in Response to Reviewers Figure 3. Note that splenic DCs were first enriched using Miltenyi CD11c magnetic beads before initiating the experiments to assess ECT.

10. Is AQP3 differentially expressed between mouse cDC1 and cDC2 ? This information can probably be found in ImmGen database and could add relevant information regarding the specialisation of cDC1 for cross-presentation.

We performed an internal analysis of AQP3 gene expression by RNAseq in CD8- and CD8+ DCs and did not observe a difference between the two populations. While one might have expected that DCs “specialized” for cross presentation would have expressed more AQP3, it is also possible that the relevant difference resides in the ability of the two populations to generate reactive oxygen species (ROS). Thus, the most efficient cross presenting DCs are able to effectively preserve antigen by maintaining the endolysosomal compartment at a higher pH during phagocytosis, partially through the spatial and temporal regulation of ROS. AQP3 appears to play a role in this process, and therefore an observed effect with AQP3 inhibition will be most prevalent in these types of DCs.

Reviewer #2: This is an elegant study on the highly important topic of antigen cross-presentation by dendritic cells of the immune system. In this study, two independent genetic screens were performed for identifying factors mediating ECT, a process well-understood to be important for antigen cross-presentation. Both screens identified the aquaporin AQP3 as a factor in ECT. A subsequent set of experiments with knockdown/rescue of AQP3, exogenously addition of H2O2, phagosomal H2O2 sensors, and mitochondrial ROS measurements then led to conclusion that mitochondria (which are positioned close to the phagosomal membrane) produce H2O2 which can diffuse through AQP3 into the lumen of mitochondria. Experiments with a lipid peroxidation probe showed that this correlates with the oxidation of phagosomal membranes. As lipid oxidation is well-known to cause membrane disruption, the authors propose a highly novel model where AQP3 translocates mitochondrial ROS into antigen-containing phagosomes which disrupts the phagosomal membrane by oxidation and thereby leads to ECT. The involvement of AQP3 in antigen cross-priming is confirmed in 2 in vivo using mouse models. The authors also show that the mitochondrial ROS production is enhanced following pathogen recognition via the adapter protein TRIF.

Altogether, the data is very convincing, and the study well designed and presented. The model proposed by the authors is radically new, and provides an explanation to the observation that large, folded proteins and other macromolecules can translocate from the lumen of endo/phagosomes into the cytosol. Of course, as with any new finding, the model also introduces several new questions that need to be addressed in future investigations, particularly why ROS have to translocate to the inside of phagosomes and cannot oxidize the phagosomal membrane from the cytosolic side. Thus, I consider this an important contribution to the antigen cross-presentation field and I expect that this study will attract a lot of attention. I recommend publication and only have some minor comments/suggestions that the authors might want to consider:

1-The actual data from the siRNA screen where AQP3 was found to mediate ECT should be shown somewhere, perhaps as a supplementary figure.

Unfortunately, this screen was conducted by a co-author (EST) at NYU and he has since left academic research and is no longer in possession of the raw data. We were encouraged that we were able to independently verify the results of the GAL4 ECT screen in our lab at Genentech. However, due to the fact that the CCF4 ECT system did not require stable expression of the UAS reporter element, all other experiments for this manuscript were performed using the CCF4 ECT system.

2-In figure 2A, it is shown that AQP2 cannot increase ECT. However, does AQP2 locate to the phagosomal membrane like AQP3? Note that while it would be good to have this information, as it shows the validity of AQP2 as a control, I consider it not strictly essential as the authors provide additional controls with the water-specific G203H mutant of AQP3 and a mutant with a disrupted dileucine motif.

While AQP2 is well known to be located on the apical membrane of cells, it also can be found on endosomes (Tajika et al., *Endocrinology*, 2004; Takata et al., *Histochem Cell Biol*., 2008). AQP2 has been shown to be stored in endosomes and upon stimulation of the hormone vasopressin, it will translocate to the apical plasma membrane, in order to facilitate water uptake from the lumen of the collecting duct (Marples et al., *Am J Physiol Cell Physiol*, 1995).

3-In figure 2E (flow cytometry) the label for the y-axis is missing.

The figure has now been updated with dsRed on the y-axis.

4-Maybe this is too speculative, but in plants aquaporins are well known to directly coordinate membrane contact sites between organelles (e.g. <https://doi.org/10.1111/nph.16743>). Maybe AQP3 itself could coordinate the contact between mitochondria and phagosomes?

This is an intriguing possibility, and thank the reviewer for calling this paper to our attention. We observed many close interactions between AQP3-decorated phagosomes and mitochondria during our EM analysis, and indeed this association has been noted previously in the literature. Absent any clear indication of physical contact, however, we thought it best not to mention this possibility. The work in the reference provided did not study phagosome-mitochondria interactions.

5-In fig S1, the authors show that the NADPH oxidase NOX2 is not involved in ECT. This contradicts previous findings from several groups. Can the authors speculate on the reason for this discrepancy?

It is possible we did not observe a role for NOX2 in our studies because we used β-lactamase to examine ECT in BMDCs. Presumably, due to the fact that it is a bacterial protein, β-lactamase was highly effective at activating DCs via TLR signaling and inducing robust ROS production. As more ROS can be made by the mitochondrial system than by the phagosomal NOX2 system, this is what we observed (recall, the mitochondria were also recruited to the vicinity of the phagosomes). While NOX2-derived ROS may therefore be irrelevant in this experimental system, our data are not necessarily incompatible with a role for NOX2. In fact, in the case of antigens that do not provide such strong innate stimuli, AQP3 and NOX2 may work in concert to increase phagosomal ROS, which ultimately results in more efficient cross presentation.

**Response to Reviewers Figures**

Figure 1

Figure 2

Figure 3
